# Coming out as an autistic researcher: academic writing and its breakdowns

**DOI:** 10.3389/fpsyt.2026.1678024

**Published:** 2026-01-30

**Authors:** Frederik Boven

**Affiliations:** Independent Researcher, Groningen, Netherlands

**Keywords:** academic writing, autistic academics, autistic researchers, autistic scholars, autistic experience, phenomenology, philosophy of autism

## Abstract

This descriptive article offers an inside perspective of the experience of writing a publishable paper by an autistic early-career researcher. From an external perspective, this experience might be described as involving hyperfocus, indecision about framing, and conflicting norms of academic writing. The article develops an inside perspective on such experiences. The author adopts a philosophical approach, using phenomenological reflection on breakdowns as a method to explicate what is implicitly given in experience. Reflection on three types of research breakdown in academic writing results in an inside description of the complexities of this particular experience by someone who is both autistic and an academic researcher.

## 1 Introduction

I am an autistic early-career researcher.

The term “autistic researcher(s)” and similar concepts emerged in academic discourse in the early 21st century to describe professional or academic researchers beyond the doctoral level who identify as autistic. They were first used to introduce openly autistic researchers such as Temple Grandin (1, 2) and Michelle Dawson (3, 4). Autistic academics have also adopted it as a self-description (5, 6). Later, the term was also used for public researchers.

In this descriptive article, I contribute to an emerging body of literature in which autistic researchers reflect on their experiences, either individually (7–11) or collectively (12–14). These authors highlight that autistic researchers face challenges, such as multitasking and networking but also possess strengths, such as pattern recognition and creativity. As my difficulties are in writing journal articles, my contribution is to reflect on this aspect of research practice.

In reflecting upon my experiences, I draw on the philosophical tradition of phenomenology (to be introduced in section two). Philosophical descriptions of the experience of autistic human beings are rare (15, 16) and within this field phenomenological investigations are only just developing (17). My investigation belongs to this emerging field and is situated at its interface with inside perspectives of autistic researchers (18).

I believe that concepts and theories of autism should not be mere applications of a general view of human cognition or functioning. Such theories cannot do justice to the complexities and subtleties of adult autistic experience. Rather, theory about autism should be wrested from our own experience as autistic individuals. I believe that when I start from my own experience, what I have to say will be more relevant to other autistic adults and other researchers as it will be more ecologically valid. I will therefore leave aside the comparative question whether my experience is similar to that of other autistic researchers or different from non-autistic researchers – questions that cannot be answered on the basis of my experience alone.

My aim is to understand the practice of academic writing from within, drawing on my experience as a writer who is both autistic and an early-career academic researcher. In pursuing this aim, my main question has been: how is my experience with academic writing structured and restructured? In this article, I will answer this question for a particular experience from my own post-doctoral career.

I will do so in four sections. The first section describes a concrete experience I had with academic writing – upon which the whole article is a reflection. Section two introduces the method I have used to reflect upon my experience: the phenomenological method of following what becomes noticeable when a field or references breaks down. In the third section, I describe the three ‘breakdowns’ of academic writing that I have reflected upon using this method. The final section discusses the results of this reflection.

## 2 An experience with academic writing

I have a Research Master’s degree in philosophy and a Ph.D. in Psychology and have published three paper in peer-review journals. Nine months after defending my dissertation, on the history of autism, I started writing a paper on Hans Asperger.

The starting point for the paper was a difference in the reception of the autism theories of Hans Asperger (19) and Leo Kanner (20): although they presented their theories around the same time (1944/1943), Kanner’s work became well known in the English language in the 1950s and 1960s, while Asperger’s work was not familiar there until the 1980s. Since Asperger wrote in German and only three of his publications have been translated in English, his work is still mostly known through the work of other authors, who rely on a selection from his writings on autism. My interest in writing the article, was clarifying how this belated and selective reception has diminished the understanding of Asperger’s conceptualization of autism.

My experience with writing this paper had three, significantly different, stages.

The *analysis stage* involved literature research and conceptual analysis, which are my main strengths. I conceptually analyzed and compared twenty papers, in German, in which Asperger described his idea of autism and thirteen papers in English, in which other authors discussed his idea before the 1980’s.

During the *framing stage*, I tried to develop the materials into a publishable article, which is my main weakness. I tried to come up with a framing of the paper that would legitimize and communicate my insights, while keeping my own goals in focus. Not knowing how to choose between possible framings, I hesitantly framed the paper as a description of the English-language reception of Asperger’s work.

During the *feedback stage*, I asked colleagues to offer feedback on the paper. Over the course of two years, I presented three subsequent drafts to my colleagues at the University of Groningen Theory and History of Psychology group. They are not autistic, work in the history of psychology and are partly more experienced researchers, whereas I am autistic, focus on the conceptual history of autism and am an early-career researcher. Each time, they responded critically, kindly pressing me to explain why my analysis was relevant to and beyond the history of autism. They concluded that the paper in its current form was not interesting enough for historians of psychology and historical journals.

After the first two feedback rounds, I experimented with alternative framings and different ways to structure the material. The feedback remained more or less the same, however. My colleagues said that with some (in their view minor) changes, the paper could become publishable. I would just have to be less exhaustive in my treatment of the sources and link up with other reception studies.

To me these changes were not minor and seemed impossible to achieve. The treatment of the sources is what interests me, so leaving much of it out made no sense to me. I struggled with placing myself in their perspective, with their different interests and idea of what is relevant. I could not organize my article according to their principle of relevance and their feedback remained too distant from what I considered relevant, a distance I could not resolve. After three attempts, I could not see a way forward that would seem acceptable to both me and other historians of psychology, and I abandoned the paper. I was confused and disoriented, unsure what had gone wrong and how I could move forward.

## 3 The phenomenological method of breakdown

In reflecting on this experience, I draw on phenomenology, a tradition founded by Husserl (21) as a descriptive psychology and later developed into a methodical philosophy (22). I take its guiding principle to be *descriptive fidelity*: “to describe what is given in experience precisely as it is given, and within the limits of how it is given” (23, p. 18). Hence, I refrain from describing experience from the outside (as in psychological experiments or comparisons) and describe experience *as I experience it.*

In developing an inside perspective on my experience of academic writing, I focus on *breakdowns:* situations in which the references to other things within the same field breaks down. I owe the term *breakdown* to Dreyfus (24). It refers to Martin Heidegger’s observation that when a hammer for example, fails to function, this is not a change in the properties of the hammer, but a breakdown of the references that allow us to experience the hammer as something ‘ready for hammering’.

In §15 of *Being and Time*, Heidegger (26) pointed out that our most familiar way of dealing with pens, door handles and knives is putting them to practical use. Heidegger’s calls all such things that we use in practice *tools.* When I use a pen *in order to* write, it is always related to other tools. My pen is related to the paper I will write on, the lamp that allows me to read in the evening, etc. Before I encounter the pen, and take it up to write something down, I have already unconsciously looked around to the other tools to which it is related. Through this meaningful whole, I know how to use the pen, what I can and cannot do with it, without thinking about it explicitly.

Ordinarily, I am not consciously aware of the tool itself and certainly not of its relations to other tools. When I use a door handle to open the door, and succeed, it is not necessary to become aware of the precise way in which my experience is structured. However, when the references ‘break down’ it often does become necessary to become consciously aware of the way my experience is structured. One such breakdown described by Heidegger is when the tool “stands in the way”, e.g. a door handle no longer opens the door. In such circumstances, one becomes aware, not just of the door handle itself, but also of the field of references that allow it to appear as a tool to be used.

Heidegger (26, p. 102-3) described such breakdown experiences as *modifications* of ordinary experience. In his view, practical involvement with the world, in which things are ‘ready for use’ is the primary way in which human beings relate to the world. When practice is interrupted, a modification of experience occurs: what now appears as an object is taken out of its practical referential context. In my intermediate case, it appeared as a concept 'ready for analysis'.

Ward turns reflection on breakdowns into a general method for the reflection on experience (25). This methods reflects on breakdown experiences to develop implicit knowledge into explicit understanding (25). I have followed this method: I started from implicit knowing, I phenomenologically reflected on my experiences, and then explicitly conceptualized it.

## 4 Three forms of ‘breakdown’ in academic writing

Ward (22) shows that Heidegger not only described *tool* breakdowns, but also *social* and *meaning* breakdowns. In my experiences with academic writing, did I also experience these three types of breakdown? Yes and no. Yes, I had experiences in which certain references to a wider field are interrupted and which are in that sense ‘breakdowns’. No, they were different from the kind described by Heidegger and Ward. This means that I have to modify their concepts somewhat to make them fit my experience.

First, when I did conceptual analysis for my paper, I often experienced that something *stood in the way* of what I wanted to do, as in tool breakdowns. However, rather than experiencing a break in my practical involvement with the world, I experienced physical things as obstacles precisely because they require practical involvement. I experienced them as obstacles I needed to concern myself with first, before I could return to my passionate interest. I experienced my clothes as an obstacle when I had to do the laundry, my computer when it started up too slowly and even my own body when I needed to sleep to keep it healthy. They resemble tool break downs, but in a sense they are the opposite: the references that broke down were not practical but rather conceptual.

Reflection on this ‘breakdown’ made me aware that my experience while doing conceptual analysis was different from my experience of getting groceries or cooking, which are the rare practices in which I am fully practically involved with the world. In both cases. my experience is structured as a ‘field’ but with a different principle of relevance. In the supermarket, I am only interested in getting the products I can use while cooking. I experience as relevant only the tools *I could use*. By contrast, when I do conceptual analysis, my interest in reading and writing about Asperger’s concept of autism is not governed by the way I could use this information. I experience things as interesting because they are *conceptually related* – other things seem useless distractions. This allows me to work very focused and stay with a topic until I really mastered it. However, many such focused investigations are detours that in the end do not contribute to what I am writing.

Second, when I tried to develop the results of my analysis into a publishable paper I tried to imagine what would be useful to my readers, but I was so absorbed in the conceptual field in which my focused interest was embedded that no other field of reference seemed to make sense to me. I could think of several possible more practical framings, but I could not really place myself within them, and none of them stood out to me as particularly relevant or interesting. I kept switching back and forth between them, until the whole framing exercise to me seemed meaningless and arbitrary. My writing no longer seemed meaningful *to me*. This resembles a *meaning ‘breakdown’* but in a different way: it was not a loss of practical meaning but rather a loss of meaning derived from a field of conceptual references.

My reflection on this meaning ‘breakdown’ made me aware that the unedited results of my analysis were very meaningful to me. They derived their meaning from a ‘field’ that was much larger than the work of Asperger: his concepts were related to concepts used by other authors. He borrowed the concept of ‘integration’ from one author and criticized the concept of a ‘lack of feeling’ used by another author. The concept I focused on was at the center of a field that included the whole conceptual context of the concept. Having this context often works to my advantage, when I see conceptual connections that others do not see, or understand the meaning of a concept on a different level. When it comes to publishing papers, it often works against me, because I find myself unable to embed my research within a more pragmatic field of concerns. My work *becomes* meaningless when it is lifted out of the field of relations that give it meaning and I cannot re-interpret it more pragmatically.

Third, on each of the three occasions my colleagues gave feedback on my paper, I rather felt like I had to integrate within a group where I was an outsider from the start. My colleagues did not engage with what I had found out, the results of my conceptual analysis, which for me was very meaningful and the core of the paper Instead, they asked what problem the paper solved, what question it answered and what is gained by showing that major aspects of Asperger’s work have not been taken up by other authors. During the discussion, and for months after, I contemplated their kind suggestions, which seemed reasonable, but didn’t seem to be options *for me*. I experienced something similar to *social* breakdown, but, again, in a different way: it was not a breakdown of shared social norms, but of my own implicit norms for academic writing.

In reflecting on this last type of ‘breakdown’, I realized that research for me is not a pragmatic activity. It is not a tool that I use in order to achieve *something practical*. I do research because I am intensely interested in something and want to find out all about it. Because it does not have a goal, my interest has no end. I read and write because it is the activity I default to if nothing stands in its way. I experienced a clash of two ‘fields’: the field in which research derives its meaning from a pragmatic field of references and research which derives its meaning from a conceptual field of references. I oscillated between these two fields, without being able to resolve the distance and the tension between them and making them overlap. This can be my strength, as it can lead to discoveries, but it can also easily lead to detours that do not yield any relevant result.

## 5 Results

All in all, how do I experience academic writing as a writer who is both autistic and an early-career researcher? Reflecting on my experience, I came to realize three things. First, while pursuing one of my intense interests, I am interested in things as they are ‘ready to analysis’ not as they are ‘ready to be used’. Second, for me, things that are ‘ready to analysis’ are also embedded within a frame of references, but of a conceptual rather than a practical kind. Third, for me, analyzing things is the most familiar and most pervasive way of relating to the world, which ‘break downs’ when it is interrupted by practical demands.

The descriptive research reported here is idiographic and limited to my own experience, but it does suggests two things. First, experiencing things in the world as ‘ready to analysis’ rather than as ‘ready to be used’ can for a human being be the most familiar and pervasive mode of relating to other beings in the world. Second, ‘breakdowns’, in the sense of interruptions of this analytical mode, can help to become aware of the structure and restructuring of such experience. Third, when I am intensely focused on a single interests, the objects of my interest is not given in isolation but as related to other aspects of experience within a field. My experience was constantly *restructured* in what may be called *field dynamics*.

How do these results relate to similar phenomenological work on autism? Reflection on these different ‘breakdowns’ confirms that different aspects of experience are *structured* within *fields*: relational wholes that give meaning and structure to aspects of experience with the field. The results of my analysis of Asperger’s papers became meaningful to me in relation to other concepts. That my final draft had a different meaning to my colleagues than it had to me shows that what seems the same phenomenon can have a different meaning in different fields. Other phenomenologists reflecting on autistic experience have described something similar, using different terms, such as ‘lifeworld’ (27), ‘autistic bubbles’ (28), ‘figure/ground structure’ (29) and ‘bi-personal fields’ (30).

Reflection on these three ‘breakdowns’ further confirms that fields are not static but are continually shifting. That the results of my conceptual analysis became meaningless, shows that phenomena can be lifted out of a field. Other phenomenologists reflecting on autistic experience have described something similar to what I call the dynamics within and between fields in terms of ‘hypnotic shifts’ (31), ‘existential-semiotic movements’ (32) and ‘bodily disorientation’ (33).

Additional research is needed to develop the phenomenological approach to autism further, apply it to autistic experience more rigorously and extensively, and use it to criticize conceptualizations of autism based on external rather internal investigations of autistic experience.

## Data Availability

The original contributions presented in the study are included in the article/supplementary material. Further inquiries can be directed to the corresponding author.
